# A High-Dynamic-Range Switched-Capacitor Sigma-Delta ADC for Digital Micromechanical Vibration Gyroscopes

**DOI:** 10.3390/mi9080372

**Published:** 2018-07-27

**Authors:** Risheng Lv, Weiping Chen, Xiaowei Liu

**Affiliations:** 1 MEMS Center, Harbin Institute of Technology, Harbin 150001, China; lvrisheng@hit.edu.cn (R.L.); weipingchen.hit@gmail.com (W.C.); 2 Key Laboratory of Micro-Systems and Micro-Structures Manufacturing (Harbin Institute of Technology), Ministry of Education, Harbin 150001, China; 3 State Key Laboratory of Urban Water Resource and Environment, Harbin Institute of Technology, Harbin 150001, China

**Keywords:** MASH ADC, sigma-delta modulation, digital noise cancellation logic, multi-rate decimator, MEMS digital gyroscopes

## Abstract

This paper presents a multi-stage noise shaping (MASH) switched-capacitor (SC) sigma-delta (ΣΔ) analog-to-digital converter (ADC) composed of an analog modulator with an on-chip noise cancellation logic and a reconfigurable digital decimator for MEMS digital gyroscope applications. A MASH 2-1-1 structure is employed to guarantee an absolutely stable modulation system. Based on the over-sampling and noise-shaping techniques, the core modulator architecture is a cascade of three single-loop stages containing feedback paths for systematic optimization to avoid deterioration in conversion accuracy caused by capacitor mismatch. A digital noise cancellation logic is also included to eliminate residual quantization errors in the former two stages, and those in the last stage are shaped by a fourth-order modulation. A multi-rate decimator follows the analog modulator to suit variable gyroscope bandwidth. Manufactured in a standard 0.35 μm CMOS technology, the whole chip occupies an area of 3.8 mm^2^. Experimental results show a maximum signal-to-noise ratio (SNR) of 100.2 dB and an overall dynamic range (DR) of 107.6 dB, with a power consumption of 3.2 mW from a 5 V supply. This corresponds to a state-of-the-art figure-of-merit (FoM) of 165.6 dB.

## 1. Introduction

**Figure 1:**
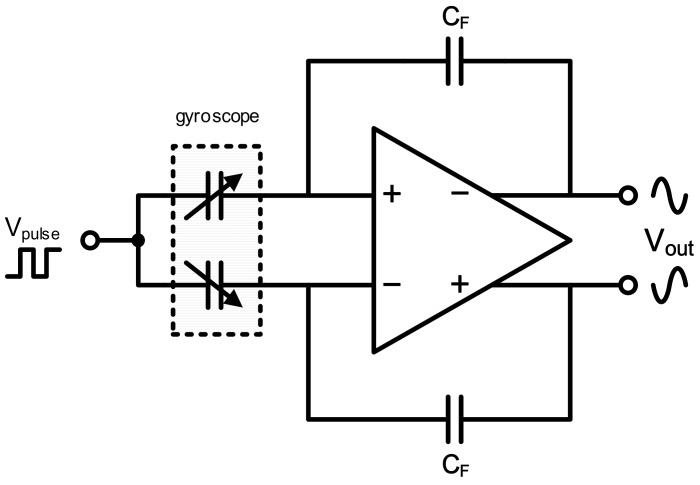
C/V conversion stage in gyroscope interface Application Specific Integrated Circuits (ASICs).

Over the last decade, a significant increase in both research effort and commercial products has occurred in the field of MEMS inertial sensors distinguished by low cost, small size, low power consumption, and suitability for batch fabrication [1]. MEMS gyroscopes, as one of the most widely used inertial sensors, are employed in a wide array of applications ranging from automotive to industrial and consumer electronics [2]. Recently, the possibility of MEMS digital gyroscopes has generated wide interest in both micromechanical sensitive structures and corresponding interface circuits [3,4,5,6,7]. Among MEMS gyroscope designs, the most investigated gyroscopes are vibratory rate gyroscopes. Generally, the structure of a typical micromechanical vibratory rate gyroscope has at least 2-DOF (degree-of-freedom) motion capability to achieve a Coriolis induced energy transfer between two separate resonance modes (drive and sense), even in a recent design based on novel principle and material [8,9]. A standard digital gyroscope is controlled by a digital closed loop, which is on the basis of a remarkable analog-to-digital converter (ADC). Specifically, the micromechanical sensitive structure and analog former circuit compose the charge amplifier to realize C/V conversion, as shown in Figure 1. This realizes capacitance detection which is widely used as a bridge from mechanical properties to electrical ones in most gyroscope designs [10,11]. This means capacitor variation resulted from mechanical motion in gyroscopes is thus transformed into voltage signals. Following ADCs convert these sinusoidal signals representing angle variation into digital forms for subsequent processing to realize practical functions. This is obviously beneficial to overall bias instability of gyroscopes due to the anti-interference capacity to temperature and processing simplicity of digital signals. Therefore, accurate data conversion becomes one of central issues in the field of ASICs (Application Specific Integrated Circuit) for high-precision MEMS digital gyroscopes. Despite different systematic controlling methods, high-precision ADCs are necessary in implemented circuits for digital gyroscopes [7,12,13,14].

A key technique of data conversion is ΣΔ modulation, a common combination of over-sampling and noise-shaping techniques. In consequence, high resolution and linearity are provided by ΣΔ ADCs. Digital post processing is also used, making it possible to reconfigure the converter for various specifications. Furthermore, oversampling ADCs do not need steep roll-off anti-alias filtering typically required in Nyquist-rate ones [15]. It is a known fact that a multi-level quantizer decreases quantization errors and results in a lower noise floor. Extra dynamic-element-matching (DEM), however, is needed for linearity improvement and also increases circuitry complexity [16]. In prior ADC designs with similar bandwidth, medium conversion accuracy is achieved by other architectures for power efficiency in different applications [17,18,19]. An architecture of shared integrators has been also proposed for low power and small size [20]. A dynamic zoom ADC proposed a remarkable combination of two conversion methods (SAR and ΣΔ) for audio applications aiming at low distortion and a 20 kHz signal band [21]. Another design presented in Ref. [22] employed inverter-based integrators for power efficiency and offset reduction, but was restricted to DC conversion. Nevertheless, a specific ADC with relatively low bandwidth and high precision for gyroscope signals has still been lacking attention. Among different realizations of ΣΔ modulators, the MASH architecture stands out for its advantages in characteristic stability and high dynamic range compared with single loop schemes, which allows a wide input range of gyroscopes.

In this paper, a MASH 2-1-1 ΣΔ ADC with a high dynamic range specialized for MEMS digital gyroscopes is presented. Feedback paths are also employed for insensitivity to component mismatch caused by the MASH architecture. Single-bit quantizers are chosen in the analog modulator. Except for inherent linearity, they also help the outputs of integrators get rid of input effects. In this work, on-chip noise cancellation logic is used to remove residual quantization noise in former analog stages. Out-of-band noise is further filtered by a digital decimator. Adjustable bandwidths are also achieved by flexible decimation ratio for actual applications. Manufactured in standard 0.35 μm CMOS technology, this work achieves 107.6 dB DR over a 2 kHz bandwidth and dissipates 3.2 mW.

This paper is organized as follows. Section 2 illustrates the systematic architecture. Implementation considerations of the analog modulator and digital decimator are adequately described in Section 3 and Section 4, respectively. Experimental results are shown and discussed in Section 5, and conclusions are finally drawn in Section 6.

## 2. System Modeling and Mismatch Analysis

**Figure 2:**
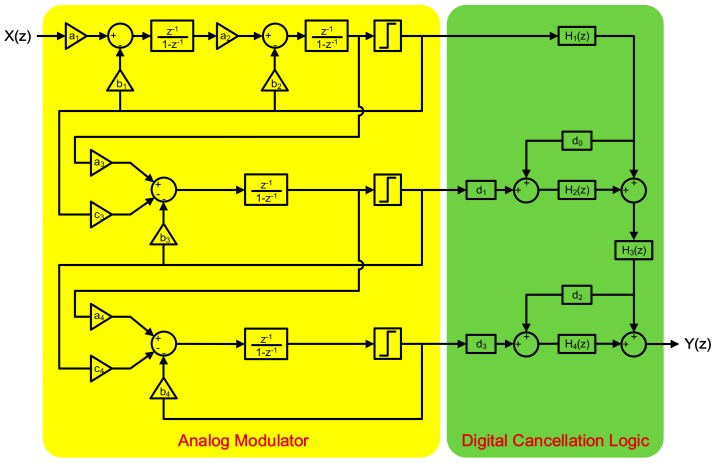
Proposed MASH 2-1-1 ΣΔ modulator architecture.

In high-accuracy applications, non-idealities resulting from circuits lead to decrease in output precision. Therefore, coefficients between stages are employed in this work to decrease signal ranges for higher linearity, lower power consumption, and lower possibility of quantizer overload. As a result, a one-bit internal quantizer is enough for this design, and prevents complexity and power dissipation of a multi-bit quantizer with corresponding dynamic element matching (DEM) or any other linearization technique introduced in Ref. [4]. Possibilities of quantizer overload are incidentally weakened. Moreover, optimization of feedback paths is also realized so that nearly only quantization errors remain in modulation loop, which significantly limits signal ranges. The system level view of the proposed MASH 2-1-1 modulation system is illustrated in Figure 2, and gain coefficients are listed in Table 1. Consisting of an analog modulator and digital cancellation logic, this circuitry structure can offer a serial digital output with most residual quantization error suppressed. In order for explicit expression, *STF* and *NTF* are used here representing signal transfer function and noise transfer function, respectively. Based on the proposed modulation architecture above, the output of first stage is expressed as (1), where *X*_1_(*z*) stand for input signal, and *Y*_1_(*z*), *E*_1_(*z*), *q*_1_ for output, quantization error, quantizer gain, respectively.

(1) $$Y_{1}(z)=STF_{1}(z)\cdot X_{1}(z)+NTF_{1}(z)\cdot E_{1}(z)=\frac{a_{1}a_{2}q_{1}z^{-2}X_{1}(z)+(1-z^{-1})E_{1}(z)}{1+(b_{2}q_{1}-2)z^{-1}+(1+b_{1}a_{2}q_{1}-b_{2}q_{1})z^{-2}}$$

In addition, *STF* and *NTF* are calculated as

(2) $$STF_{1}(z)=\frac{a_{1}a_{2}q_{1}z^{-2}}{1+(b_{2}q_{1}-2)z^{-1}+(1+b_{1}a_{2}q_{1}-b_{2}q_{1})z^{-2}}$$

(3) $$NTF_{1}(z)=\frac{1-z^{-1}}{1+(b_{2}q_{1}-2)z^{-1}+(1+b_{1}a_{2}q_{1}-b_{2}q_{1})z^{-2}}$$

For a second order noise shaping capacity in the first stage of the MASH 2-1-1 modulation system, Equations (4) and (5) must be satisfied as follows.

(4) $$b_{2}q_{1}-2=0$$

(5) $$1+b_{1}a_{2}q_{1}-b_{2}q_{1}=0$$

As a result, Equation (1) can be rewritten as

(6) $$Y_{1}(z)=\frac{a_{1}}{b_{1}}z^{-2}X_{1}(z)+(1-z^{-1})E_{1}(z)$$

Similarly, outputs of the second and third stage are given by Equations (7) and (8) without a detailed calculation, where *X_i_*(*z*), *Y_i_*(*z*), *E_i_*(*z*), *q_i_* (*i* = 2,3) represent inputs, outputs, quantization errors, and quantizer gains of latter stages, respectively.

(7) $$Y_{2}(z)=STF_{2}(z)\cdot X_{2}(z)+NTF_{2}(z)\cdot E_{2}(z)=\frac{a_{3}}{c_{3}}z^{-1}X_{2}(z)+(1-z^{-1})E_{2}\left(z\right)$$

(8) $$Y_{3}(z)=STF_{3}(z)\cdot X_{3}(z)+NTF_{3}(z)\cdot E_{3}(z)=\frac{a_{4}}{c_{4}}z^{-1}X_{3}(z)+(1-z^{-1})E_{3}(z)$$

On the basis of outputs of stages in the analog modulator, digital cancellation logic can be analyzed accordingly. First of all, the final output should only be in relation with initial input and quantization errors. On the assumption that the final digital output of noise cancellation logic is a function of outputs of each stage, it also contains quantization errors, as follows

(9) $$Y(z)=H_{1}(z)H_{3}(z)[1+d_{0}H_{2}(z)][1+d_{2}H_{4}(z)]\cdot Y_{1}(z)+d_{1}H_{2}(z)H_{3}(z)[1+d_{2}H_{4}(z)]\cdot Y_{2}(z)+d_{3}H_{4}(z)\cdot Y_{3}(z)$$

It is a main consideration that MASH modulation architecture uses a digital algorithm to eliminate a major proportion of quantization errors, with only those in the last stage suppressed by high-order modulation. For entire cancellation of former quantization errors, namely *E*_1_(*z*) and *E*_2_(*z*), they are designed to attenuate to zero. Thus, we have the final form of the digital output *Y*(*z*) in Equation (10) and *H_i_*(*z*) (*i* = 1,2,3,4) in Equations (11)–(14)

(10) $$Y(z)=\frac{a_{1}}{b_{1}}z^{-4}\cdot X(z)+d_{3}{\left(1-z^{-1}\right)}^{4}\cdot E_{3}(z)$$

(11) $$H_{1}(z)=z^{-1}$$

(12) $$H_{2}(z)={\left(1-z^{-1}\right)}^{2}$$

(13) $$H_{3}(z)=z^{-1}$$

(14) $$H_{4}(z)={\left(1-z^{-1}\right)}^{3}$$

Obviously, the final digital output depends only on initial input and a fourth-order modulated quantization error *E*_3_(*z*).

As illustrated above, a MASH modulation architecture suffers generally from mismatch between analog and digital circuits. This mainly comes from capacitor mismatch, i.e., differences between ideal gain coefficients and actual implementation values, which are commonly realized by unit capacitor ratios. Due to manufacturing errors and process factors, this is almost unavoidable. However, there are still some methods of decreasing influences resulting from these unideal properties. Especially, restricting integrator output swings is a reasonable way. Since these voltage amplitudes are reduced, tiny gain errors due to mismatch can be less obvious. Besides, one-bit quantization also weakens this influence on account of mismatch in feedback digital-to-analog converter (DAC). On the basis of progressive comprehension of the implemented manufacturing technology, most process deviation can be quantified or at least estimated. Proper modification of coefficients in digital cancellation logic is available accordingly, which also helps solve this problem.

With deviation taken into consideration, the coefficients differ slightly from their design values, shown as Equations (15)–(17). A concept of error factor ε is introduced here.

(15) $$a_{i,p}=a_{i}(1+\varepsilon_{a_{i}}),\;i=1,2,3,4$$

(16) $$b_{i,p}=b_{i}(1+\varepsilon_{b_{i}}),\;i=1,2,3,4$$

(17) $$c_{i,p}=c_{i}(1+\varepsilon_{c_{i}}),\;i=3,4$$

According to Equations (15)–(17), we can get a new expression of the final digital output *Y*(*z*) with high order terms omitted, as shown in Equation (18)

(18) $$Y(z)=\frac{a_{1}}{b_{1}}z^{-4}X(z)-z^{-2}{\left(1-z^{-1}\right)}^{2}\sum_{i=1}^{4}\varepsilon_{a_{i}}+d_{1}{\left(1-z^{-1}\right)}^{4}E_{2}(z)$$

Assuming quantization error is white, in-band power of quantization error can be expressed as Equation (19), where Δ is quantization step. And *OSR* denotes the oversampling ratio expressed by $OSR=f_{s}/2BW$, where *f_s_* is sampling frequency and *BW* is signal bandwidth.

(19) $$P_{E}=\frac{\Delta^{2}}{12}[\frac{\pi^{4}}{5OSR^{5}}{\left(\sum_{i=1}^{4}\varepsilon_{a_{i}}\right)}^{2}+\frac{2\pi^{8}}{9OSR^{9}}d_{1}^{2}]$$

Therefore, overall DR can be expressed as

(20) $$\mathrm{DR}={\left[\frac{2\pi^{4}}{15OSR^{5}}{\left(\sum_{i=1}^{4}\varepsilon_{a_{i}}\right)}^{2}+\frac{2\pi^{8}}{27OSR^{9}}d_{1}^{2}\right]}^{-1}$$

Deterioration in DR due to mismatch can be calculated as

(21) $$\Delta \mathrm{DR}=1+\frac{9OSR^{4}}{5\pi^{4}d_{1}^{2}}{\left(\sum_{i=1}^{4}\varepsilon_{a_{i}}\right)}^{2}$$

According to the above calculation, quantization errors in the former two stages are not completely eliminated by digital cancellation logic and leak to the final output. As a result, in-band noise power increases, thus leading to a reduction in DR. Generally, quantization error leakage counts most in the first stage. A second order modulation is consequently employed in this work for leakage suppression.

As a cascade of former analog modulator and latter digital decimator, an ADC is constructed according to actual matching requirements. First, the signal bandwidth of the digital decimator is by no means less than that of the former analog modulator. Otherwise, the overall ADC signal bandwidth decreases and the input signal may be attenuated. An excessive signal bandwidth of digital decimator is also adverse as well, for a portion of high-frequency noises may remain unfiltered which act as jitter in the time domain.

**Figure 3:**
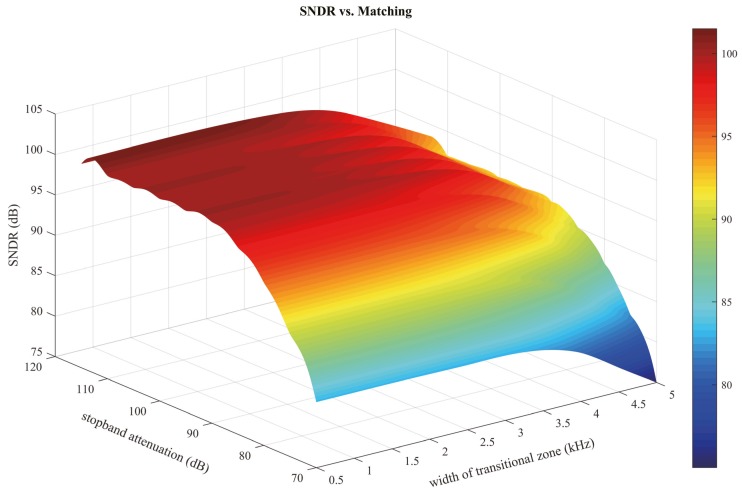
Simulation results indicating tendencies between signal to noise and distortion ratio (SNDR) and matching.

Additionally, the indexes of stopband attenuation and the width of transitional zone must be carefully chosen to insure no extra noise is folded into the signal band during digital decimation. A wide transitional zone and an insufficient stopband attenuation can result in residual noises being mixed into the signal band and relevant resolution loss. Harsh restrictions, however, are not a requisite either, in that they may cost unacceptable area and power wastage. Based on simulations of combinations of different matching parameters, a balanced choice is obtained and the implemented ADC is designed accordingly. Simulation results in Figure 3 show a signal to noise and distortion ratio (SNDR) of 99.8 dB with a 90 dB stopband attenuation and 3 kHz transitional zone.

## 3. Analog Modulator Implementation

**Figure 4:**
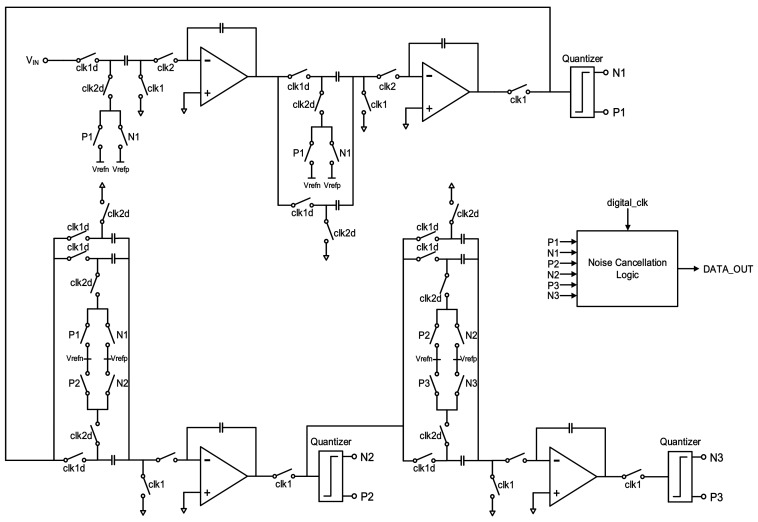
Simplified schematic of the proposed modulator.

The modulation system is analyzed above and is designed to be implemented by fully differential topology accordingly for its common-mode noise elimination, high power supply noise rejection, and even order harmonic distortion extermination. In addition, an extra 3 dB higher SNR than single-ended realization is another advantage. Figure 4 gives a simplified schematic diagram. All integrators are based on a parasitic-insensitive design to get rid of performance decrease due to charge injection in CMOS switches.

A high-accuracy ΣΔ modulator is remarkable for its high linearity, which is fundamental for precise data conversion. In consequence, fully differential amplifiers are necessary for low noise and distortion, especially in the first integrator. A MASH modulation architecture relaxes the requirements for amplifiers due to its superiority of absolute stability. A similar noise shaping capacity between a MASH and single-loop architecture is obvious on the assumption of the same modulation order. Operating at the same sampling frequency, however, the MASH architecture shows looser constraints on oversampling ratio (OSR) than a single-loop substitute. This means a relatively lower sampling frequency is available without any deterioration of resolution. Dynamic performances for amplifiers (e.g., gain-bandwidth product and slew-rate) are therefore relaxed, which is also advantageous for power economization.

**Figure 5:**
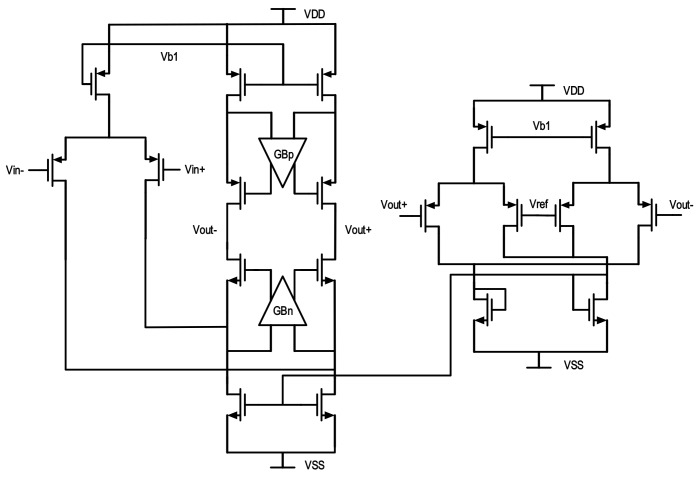
Fully differential amplifier in the first integrator.

In this work, a single-stage, folded-cascode, gain-boosting amplifier is designed in the first integrator, which is proved to have dominant impacts on the performance of the ΣΔ modulator, especially on harmonic distortion. Compared with a multi-stage amplifier, this choice is competitive in high-speed applications. Figure 5 shows the fully-differential gain-boosting amplifier used in the first integrator with a continuous-time common-mode feed-back circuit. It is quite obvious that the equivalent noise at the input of the first integrator mixes directly with the final output within the signal bandwidth, while that of other integrators are shaped increasingly. Hence, the first integrator needs especial attention and are specially designed for low in-band white noise. Its gain, bandwidth and slew rate will determine integration setup accuracy and result in distortion. Chopper stabilization is also applied to suppress flicker noise within the signal band, while white noise remains unaffected. The input signal is chopped ahead by half of the sampling frequency, i.e., *f_s_*/2, so that flicker noise is modulated to high frequency centered at *f_s_*/2, and filtered out later. As a result, the noise floor in the signal bandwidth is largely suppressed. For the remaining amplifiers in latter integrators, simple telescopic cascode ones are implemented.

Additionally, the equivalent capacitance loads of integrators differ during sampling and integration due to switch operations, and this should also be an important consideration during amplifier design.

Now, take sampling capacitors for example. Two switches are included in a sampling path and yield a thermal-noise power spectrum density satisfying

(22) $$S^{2}(f)=8kTR_{on}$$

where *k* denotes the Boltzmann constant, *T* the absolute temperature, and *R_on_* the switch on-resistance. Practical resistances are always noisy and result in a transfer function of equivalent noise source expressed by

(23) $$H_{s}(s)=\frac{1}{1+2sR_{on}C_{S}}$$

So the equivalent noise bandwidth comes to

(24) $$BW_{s\mathrm{am}}={\int_{0}^{+\infty }\left|H_{s}(f)\right|}^{2}df=\int_{0}^{+\infty }\frac{1}{1+{\left(4\pi fR_{on}C_{S}\right)}^{2}}df=\frac{1}{8R_{on}C_{S}}$$

In order for a highly-suppressed sampling error, *BW_sam_* is generally much larger than sampling frequency *f_s_*. In consequence, aliasing occurs within signal band. During sampling, the power spectrum density of equivalent noise resulting from switches within the signal band is

(25) $$S_{in,s}^{2}(f)=\text{​}\frac{BW_{sam}}{f_{s}}\times S^{2}(f)\times Sa^{2}(\frac{\pi f}{f_{s}})=\frac{kT}{C_{S}f_{s}}Sa^{2}(\frac{\pi f}{f_{s}})\approx \frac{kT}{C_{S}f_{s}}$$

**Figure 6:**
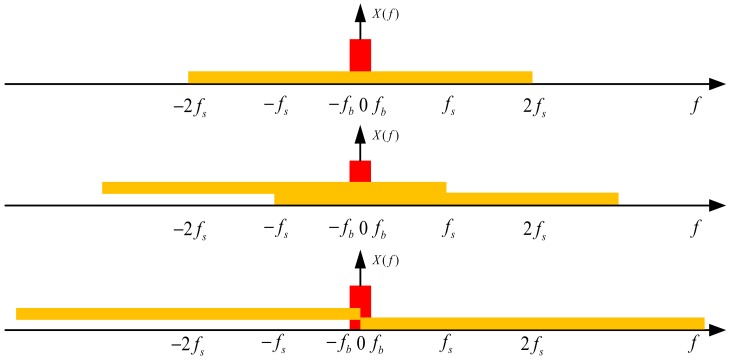
Simplified schematic of noise aliasing.

Figure 6 shows the noise aliasing in SC circuits. A double *f_s_* of *BW_sam_* is assumed, and a double noise aliasing appears within the signal band. According to (22), power spectrum density of switch noise decreases with a smaller *R_on_*. On the other hand, *BW_sam_* increases, thus introducing more noise into the signal band. However, reduced in-band noise is independent of *R_on_*, but relies on higher *C_s_* and *f_s_*. This noise adds directly to the input of first integrator and transfers to final output without any noise shaping. Actual design usually adopts an appropriately large capacitor for reducing the power spectrum density of switch noise. It is also essential to note that this also increases capacitance load and reduces setup accuracy during integration, especially for a high sampling frequency *f_s_*. Furthermore, the loop time constant decreases with a decreasing *R_on_*, thus improving sampling accuracy. But, a small *R_on_* is implemented by large transistors in which the effects of channel charge injection and clock feed-through stand out. To sum up, there is a tradeoff in the choice of *R_on_* and *C_s_*, and other circuit elements, amplifiers for instance, should be designed accordingly.

**Figure 7:**
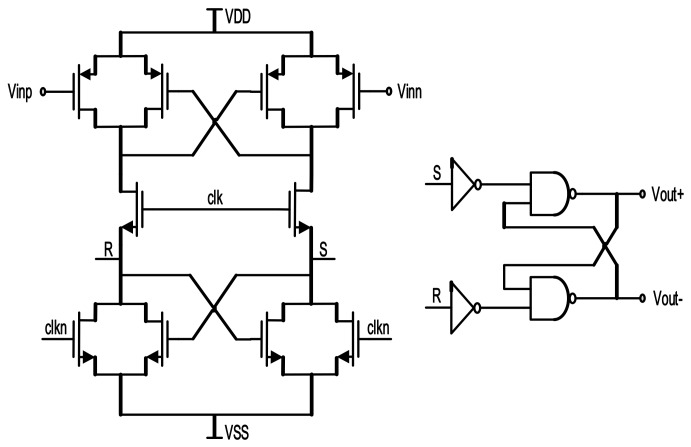
Fully differential dynamic comparator and latch.

At the beginning of each stage, we choose a passive adder composed of capacitors and switches instead of an active adder, for its high ratio of resolution to power consumption. Figure 7 shows the fully differential dynamic comparator with a CMOS latch. A ΣΔ modulation system does not demand high-speed quantization, since comparator delay is regarded as a similar effect of clock jitter. Obviously, the comparator operates when the clock is high, and holds the former state when the clock is low. For comparison precision consideration, the input transistors are set to be large in size for high voltage-resolution, while the clock-controlled transistors are designed to be small for weakened influences of charge-injection and clock-feedthrough.

**Figure 8:**
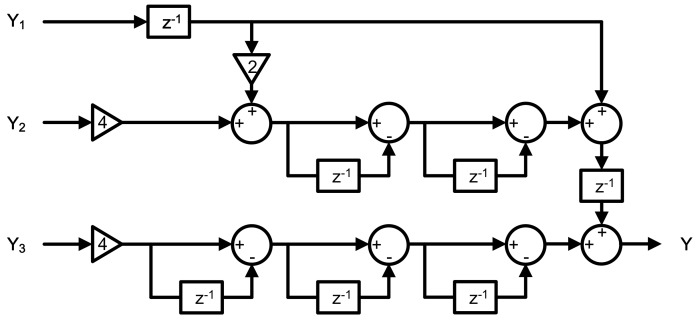
Digital noise cancellation logic architecture.

A digital cancellation logic in MASH modulators is one of main differences from high-order single-loop designs. It is used to eliminate quantization errors in the former stages. Digital cancellation logic in this work is based on the result mentioned in Table 1 and Equations (11)–(14), as shown in Figure 8. Coefficients in this logic are designed to be several times power of 2, so that they can be easily realized by a shifting operation in digital circuit realization.

## 4. Digital Filter Implementation

Former analog modulators have employed a MASH 2-1-1 architecture with the advantages of guaranteed systemic stability, simplified designing difficulties, and lower in-band noise, which is convenient for digital gyroscopes. For higher bias stabilities of the digital gyroscope sensors, the latter decimator was implemented with a cascade of CIC (Cascaded Integrator Comb) filter, CIC compensation filter, and a half-band filter. A variable decimation ratio was designed for the CIC filter for output frequency and passband cut-off frequency in support of alterable overall gyroscope bandwidths.

**Figure 9:**
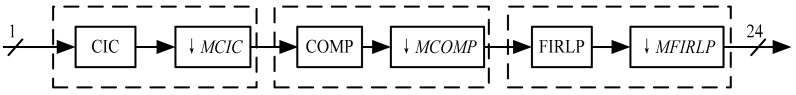
Cascade structure of digital filter.

The decimator used in this work was a cascade of three stages, and each stage was a finite impulse response (FIR) filter. Due to high performing rate in the first stage, a CIC filter was adopted for lower power consumption. In addition, due to the narrow passband and wide range of decimation ratio of a CIC filter, variable operating rates were available. However, the frequency response of a CIC filter drops gradually within passband. Therefore, a CIC compensation filter was introduced to flatten the signal band and decimate by 2. Further high frequency noise was filtered by a half-band filter with a decimation of 2. The entire architecture of the digital decimator is shown in Figure 9.

### 4.1. Modeling and Design of the CIC Filter

CIC filters are typical FIR-type filters and characteristic of fixed group delay. Multiplying units and memories are not necessary during the whole calculation.

The unit impulse response *h*[*n*] of a single stage CIC filter is

(26) $$h[n]=\{\begin{array}{ll} 1 & 0\leq n\leq D-1\\ 0 & others \end{array}$$

and the system function *H*(*z*) is

(27) $$H(z)=\sum_{n=0}^{D-1}z^{-n}=\frac{1-z^{-D}}{1-z^{-1}}$$

Supposing *x*[*n*] as an input, the output of a CIC filter is

(28) $$y[n]=x[n]*h[n]=\sum_{k=0}^{D-1}x[n-k]$$

Apparently, only an add operation is included, and average calculation.

The system function *H*(*z*) of a multi-stage CIC filter is

(29) $$H(z)={\left(\frac{1-z^{-D}}{1-z^{-1}}\right)}^{L}$$

where *D* is the length of a single stage CIC filter, and *L* defines orders.

Therefore, the magnitude–frequency response is calculated as

(30) $$20\log_{10}\left|H(\omega )\right|=20\log_{10}{\left|{\left(\frac{1-z^{-D}}{1-z^{-1}}\right)}^{L}\right|}_{z=e^{j\omega }}=L\times 20\log_{10}\left|\frac{\sin(D\omega /2)}{\sin(\omega /2)}\right|$$

where $\omega =2\pi f/f_{s}$.

According to Equation (30), frequency response is decided by *D* horizontally and by *L* vertically. As expected, passband width narrows with increasing *D*, and stopband attenuation increases with increasing *L*.

**Figure 10:**
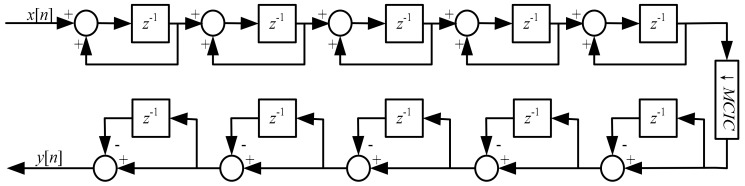
Structure of the cascaded integrator comb (CIC) filter.

Since the modulator implemented in this work is characteristic of fourth order noise shaping capacity, one more order is necessary for the CIC filter to avoid deterioration of conversion precision of the analog modulator. On the basis of the above consideration, this design adopted a five-section structure, a cascade of integrators, a decimator, and differentiators, shown in Figure 10. As a result, an alterable decimation ratio MCIC is available in order to adjust the output rate and passband edge frequency of the whole filter. Besides, the full advantages are taken of each integrator and differentiator.

The output bit width varies with the overall decimation ratio MCIC, which follows

(31) $$B_{\max}=N\log_{2}RM+B_{in}-1$$

where *N* stands for the number of sections, *R* for decimation ratio, *M* for differential delay, *B_in_* for input bit width. As a result, final output bit width must be coincident at different MCICs. In this case, MCIC converts from 32 to 4096, so a 26-bit width is chosen for the CIC output. Specific definitions are given in Table 2.

### 4.2. Modeling and Design of CIC Compensation Filter

Generally, an obvious fall exists in the CIC filter magnitude response within the signal band, and worsens with a higher order *L*. This will lead to an unexpected attenuation for input signals. Therefore, a subsequent CIC compensation filter is essential to flatten the overall filter passband, due to its characteristic magnitude response.

Thanks to the employment of an oversampling technique, the normalized frequency *ω* << 1. The approximation in Equation (32) is thus reasonable.

(32) $$\left|H(\omega )\right|={\left|\frac{\sin(D\omega /2)}{\sin(\omega /2)}\right|}^{L}\approx {\left|\frac{\sin(D\omega /2)}{\omega /2}\right|}^{L}=\left|\frac{\sin^{L}(D\omega /2)}{{\left(\omega /2\right)}^{L}}\right|$$

**Figure 11:**
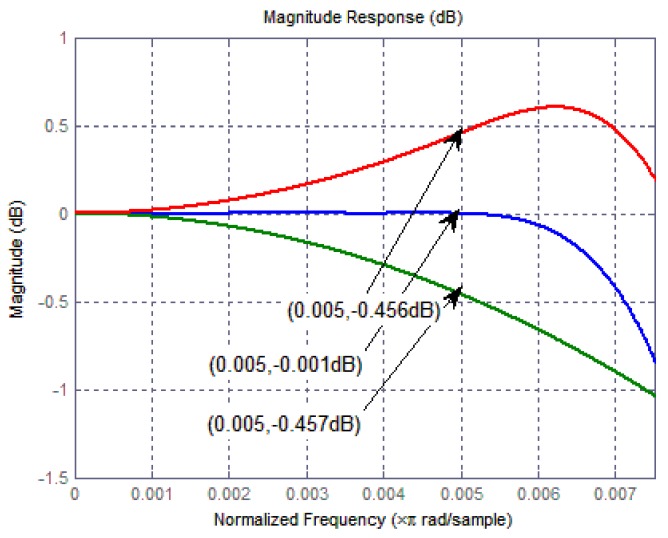
Passband magnitude responses before and after compensation.

A reciprocal form, namely

(33) $$\left|H_{comp}(\omega )\right|={\left|\frac{\sin^{L}(D\omega /2)}{{\left(\omega /2\right)}^{L}}\right|}^{-1}=\left|\frac{{\left(\omega /2\right)}^{L}}{\sin^{L}(D\omega /2)}\right|$$

describes exactly the magnitude response of a CIC compensation filter. As a result, a cascade implementation of a CIC and a CIC compensation filter provides a flat passband and avoids signal distortion. Figure 11 plots the passband magnitude as a function of normalized frequency of a CIC filter (decimation ration MCIC = 32, differential delay M = 1, section number *L* = 5), a CIC compensation filter, and the cascade of both two stages. Magnitudes before and after compensation are −0.457 dB and −0.001 dB, respectively. Moreover, a narrower transitional zone is obtained, in addition to a flat passband.

**Figure 12:**
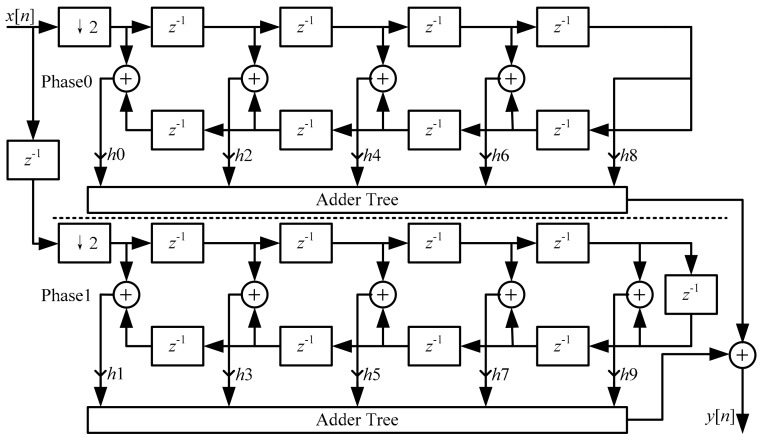
Block diagram of the optimized compensation filter.

In the practical design, a simultaneous down-sampling by 2 was implemented in the CIC compensation filter for a lower operating rate. Besides, a symmetrical filter structure was employed for reducing multipliers which saved area and power and accelerates operating clock. An optimized 18-order CIC compensation filter is described in Figure 12.

A symmetrical structure was employed in each stage in the above design on the basis of an even-order filter. With a 26-bit input, the CIC compensation filter outputs a 24-bit parallel data. The lower two bits are calculated as decimal places and participate in rounding to avoid extra zero errors.

### 4.3. Modeling and Design of the Half-Band Filter

For further suppression of high-frequency noise and another down-sampling by 2, a half-band filter followed the CIC compensation filter. As the symcenter of magnitude response is at *f_s_*/4, noise aliasing can be effectively prevented when configured as a decimation ration of 2. Noises introduced by former analog modulator in ADCs have been nearly filtered. In consequence, stopband attenuation needs to not be high for anti-aliasing.

There is still something to be done for quantization of coefficients in digital filters. The coding method has great influences on frequency response as well as circuit speed and power. All the three stages have fixed coefficients, so a CSD (Canonic Signed-Digit) code is applied for simplifying quantified coefficients. As a result, less multipliers are used and power consumption is restricted.

### 4.4. Entire Digital Filter Integration and Simulation

**Figure 13:**
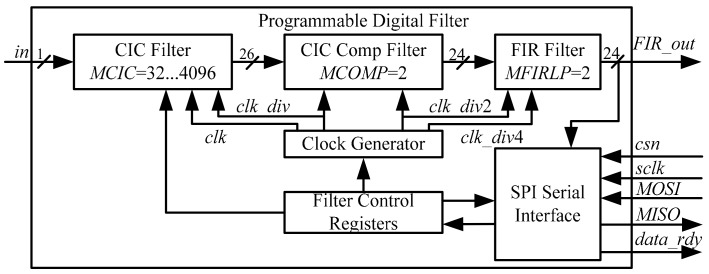
Block diagram of overall digital filter.

The whole digital filter consists mainly of clock generator, a CIC decimator, a CIC compensation filter, a half-band filter, and a SPI serial interface. Figure 13 plots the overall architecture.

**Figure 14:**
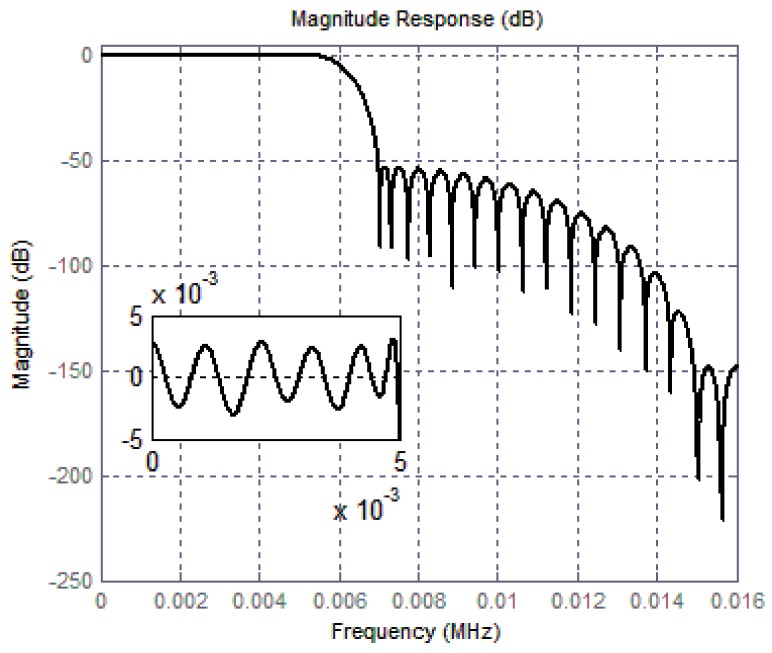
Magnitude response of the proposed digital filter.

With the operating signal band of the gyroscope sensors taken into consideration, the clock frequency for the inner ADC is generally set to several MHz. When the former modulator operates at 2048 kHz, Table 3 gives a detailed configuration for overall decimation ratio and output rate. On condition of a sampling frequency of 2048 kHz and an overall decimation ratio of 128, Figure 14 shows the magnitude response up to 16 kHz. An in-band ripple of no more than 0.005 dB is obtained and guarantees conversion accuracy.

Based on the filter performance above, decimation operation has nearly no interference in the SNR and noise floor of the preceding analog modulator. In addition, aliasing effects can be neglected.

## 5. Experimental Results and Discussion

**Figure 15:**
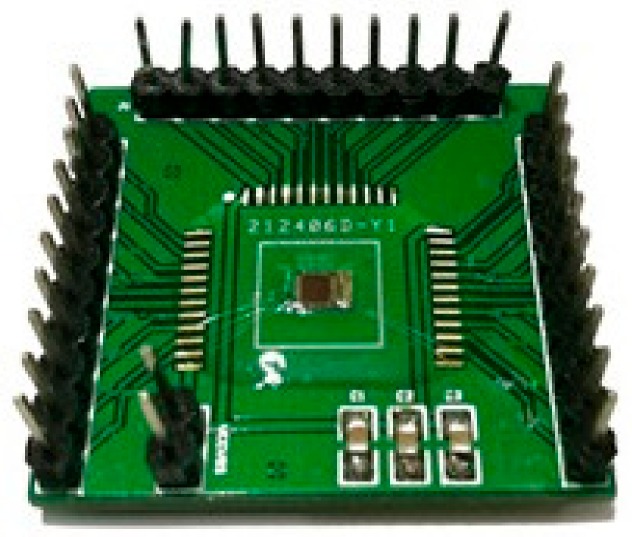
Testing chip and printed-circuit-board (PCB).

The proposed fourth-order ΣΔ ADC was fabricated in standard 0.35 μm CMOS technology. Figure 15 presents the testing printed-circuit-board (PCB) with the ADC prototype bonded. Operating at a sampling frequency of 1 MHz, the whole ADC dissipates 3.2 mW from a 5 V supply. Specifically, the digital circuitry consumes 39% of the power (non-overlapping clock generator, digital cancellation logic, and digital decimator). The first modulation stage, with its 46% share, dominates the analog power consumption. An external 5 V voltage is used as the ADC’s reference voltage. To reduce digital switching noise coupling, separate digital and analog supplies are also used. Moreover, noisy digital circuits are separated and isolated from analog circuit blocks.

**Figure 16:**
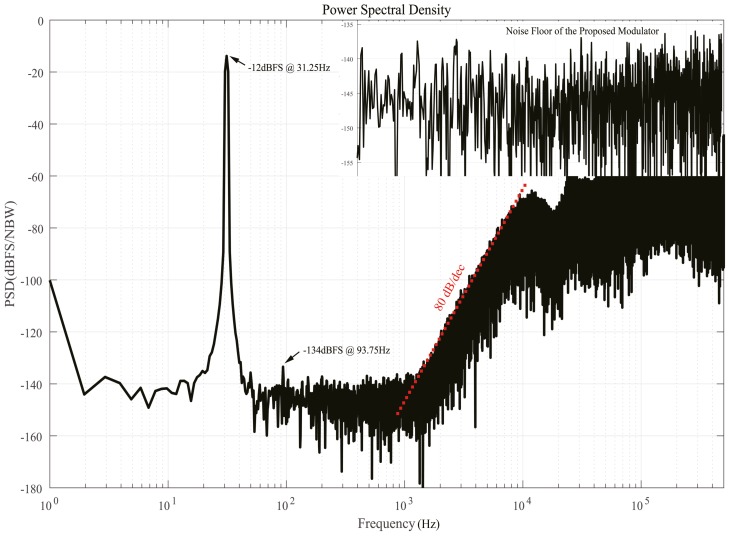
Measured modulator output spectrum.

Figure 16 shows the output spectrum of the ΣΔ modulator with a 31.25 Hz sinusoidal signal input at a sampling frequency of 1 MHz. It can be seen to be free of unexpected noises (e.g., idle tones), only power frequency interferences and the unavoidable third-order harmonic remain in signal band. A thermal-noise-limited noise of 4.3 μVrms is measured in a single conversion, resulting in an SNR of 100.2 dB for a 1.3 V differential input. The measured spurious-free dynamic range (SFDR) reaches up to 122 dB. This provides a maximum input range without any distortion. The 80 dB/dec slope of the quantization noise demonstrates that fourth-order noise shaping is achieved.

**Figure 17:**
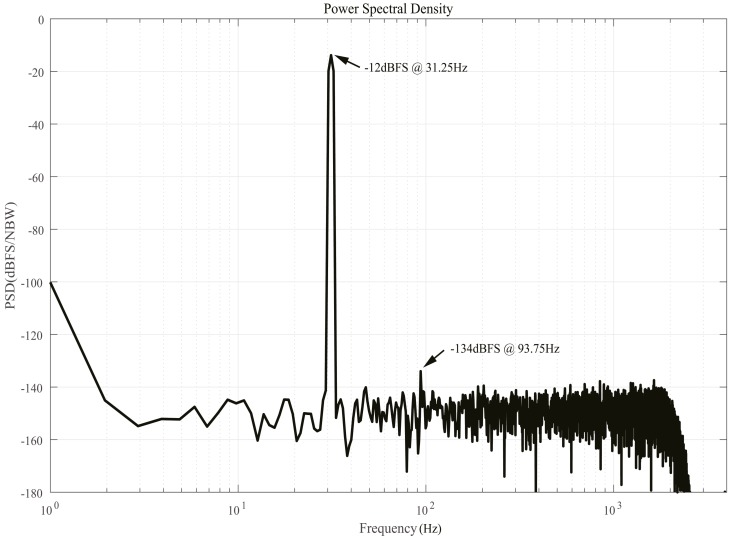
Measured ADC output spectrum.

Figure 17 shows the output spectrum of the proposed ADC, which is obtained under the same operating conditions as the analog modulator. Out-of-band noises at high frequency are filtered to insignificance. When an accumulated decimation ratio of 128 was employed, the proposed ADC presented a 2 kHz bandwidth. Moreover, other necessary bandwidths are also available by means of alterable decimation ratio for actual applications. It should also be noted that SNR and noise floor are almost constant before and after filtering, suggesting aliasing resulted from digital decimation is negligible.

In Table 4, the ADC’s performance is summarized and compared with other state-of-the-art high resolution ADCs according to a representative figure of merit (FoM) defined as

(34) $$\mathrm{FoM}={\mathrm{DR}}_{dB}+10\log(\frac{\mathrm{Bandwidth}}{Power})$$

As shown in Table 4, the listed devices [19,23,24] are the closest designs in terms of FoM to the proposed ADC. The main distinguishing factor of this work is the high dynamic range, since it uses the noise cancellation logic to suppress in-band noise. Besides, the fourth-order modulation expels most quantization noise from signal band leading to an in-band noise floor limited by thermal noises. The obtained FoM are comparable with other works in Table 4, showing that the presented ADCs are good candidates for fulfilling the application requirements of low signal band and high resolution, targeted in MEMS digital gyroscopes. In the aspect of commercial devices, parameters of a currently published ΣΔ ADC (AD7768-1) by Analog Devices in 2018 are shown in Table 4 [25]. This performance corresponds to a FoM of 171.6 dB. The ADC proposed in this work attains comparable performance and integration convenience in gyroscope interfaces.

It is also worthwhile to mention that further FoM improvement, especially in power efficiency, of the proposed ADC can be achieved by applying different enhancement techniques. As for the architecture, a dynamic zoom design [21] is a good trend. In this case, feedback references can be adjusted and optimized promptly according to the current input signal level, and therefore loosen the accuracy requirements for the analog modulator. Besides, the index of DR will incidentally rise due to quantization error reduction. From the circuit implementation perspective, state-of-the-art power-efficient circuit techniques, such as inverter-based integrators [22], can be employed to take better advantage of the relaxed circuit specifications, particularly in the first stage.

## 6. Conclusions

This paper describes a MASH ADC digitizing gyroscope signal with state-of-the-art conversion precision and FoM. Additionally, the use and effectiveness of reconfigurable decimator following modulators are investigated, with special focus on various bandwidth applications, particularly for ADCs in universal inertial sensor devices such as gyroscopes. To provide an accurate and stable solution for the required A/D conversion, a MASH 2-1-1 modulation architecture, consisting of three stages in a cascade configuration, has been proposed. This architecture employs a combination of feedback paths and single-bit quantization to reduce voltage swings in integrators and to improve linearity. The sensitivity of a MASH architecture to capacitor mismatch has been largely attenuated by special design both in circuit and layout. A decimator with adjustable decimation ratio has been demonstrated in detail. The prototype ADC has been fabricated in standard 0.35 μm CMOS technology. From a 5 V supply, the achieved 107.6 dB and 165.6 dB of DR and FoM make the proposed ADC one of the most impressive designs among the published low speed ADCs.

## Figures and Tables

**Table 1:** Coefficients of the proposed modulator.

Coefficient	*a*_1_	*b*_1_	*a*_2_	*b*_2_	*a*_3_	*b*_3_	*c*_3_
**Value**	0.25	0.25	0.5	0.25	1	0.5	0.5
**Coefficient**	a_4_	b_4_	c_4_	d_0_	d_1_	d_2_	d_3_
**Value**	1	0.5	0.5	2	4	0	4

**Table 2:** Data truncation at different MCICs.

MCIC	CIC Output
32	data[25:0]
64	data[30:5]
128	data[35:10]
256	data[40:15]
512	data[45:20]
1024	data[50:25]
2048	data[55:30]
4096	data[60:35]

**Table 3:** Detailed configuration for main indexes (f_in_ = 2048 kHz).

Decimation Ratio	Output Rate (Hz)
128	16,000
256	8000
512	4000
1024	2000
2048	1000
4096	500
8192	250
16,384	125

**Table 4:** Performance summary and comparison.

Reference	[25]	[23]	[20]	[24]	[19]	[26]	[27]	[28]	This Work
**Year**	2018	2017	2016	2016	2015	2013	2011	2009	2017
**Bandwidth (kHz)**	13.9	7.5	156.25	0.3	4	2	10	1.56	2
**SNR (dB)**	107.8	60.5	57.7	85	75.9	65.3	61	65.63	100.2
**DR (dB)**	108.5	70.1	-	88	85.5	68.2	64	69	107.6
**Supply (V)**	5	0.4	1.8	1	1.2	1.6	0.25	11.2	5
**Power (mW)**	6.75	0.0127	0.0295	0.023	0.0348	0.096	0.0075	63.3	3.2
**Technology (μm)**	-	0.065	0.18	0.18	0.18	0.15	0.18	3	0.35
**FoM (dB)**	171.6	157.8	154.9	159.2	166.1	141.4	155.2	112.9	165.6
